# Conformational flexibility of EptA driven by an interdomain helix provides insights for enzyme–substrate recognition

**DOI:** 10.1107/S2052252521005613

**Published:** 2021-07-15

**Authors:** Anandhi Anandan, Nicholas W. Dunstan, Timothy M. Ryan, Haydyn D. T. Mertens, Katherine Y. L. Lim, Genevieve L. Evans, Charlene M. Kahler, Alice Vrielink

**Affiliations:** a School of Molecular Sciences, The University of Western Australia, 35 Stirling Highway, Perth 6009, Australia; b Australian Synchrotron, 800 Blackburn Road, Clayton, Victoria 3168, Australia; c European Molecular Biology Laboratory, Hamburg Unit, DESY, Notkestrasse 85, 22607 Hamburg, Germany; d The Marshall Centre for Infectious Diseases Research and Training, School of Biomedical Sciences, University of Western Australia, 35 Stirling Highway, Perth, Western Australia 6009, Australia

**Keywords:** EptA, phosphoethanolamine transferase, small-angle X-ray scattering, tryptophan fluorescence, conformational flexibility, enzyme substrate recognition

## Abstract

Many pathogenic gram-negative bacteria have developed mechanisms to increase resistance to cationic antimicrobial peptides by modifying the lipid A moiety. One modification is the addition of phospho­ethano­lamine to lipid A by the enzyme phospho­ethano­lamine transferase (EptA). Previously we reported the structure of EptA from *Neisseria*, revealing a two-domain architecture consisting of a periplasmic facing soluble domain and a transmembrane domain, linked together by a bridging helix. Here, the conformational flexibility of EptA in different detergent environments is probed by solution scattering and intrinsic fluorescence-quenching studies. The solution scattering studies reveal the enzyme in a more compact state with the two domains positioned close together in an *n*-do­decyl-β-d-maltoside micelle environment and an open extended structure in an *n*-do­decyl-phospho­choline micelle environment. Intrinsic fluorescence quenching studies localize the domain movements to the bridging helix. These results provide important insights into substrate binding and the molecular mechanism of endotoxin modification by EptA.

## Abstract 2

Small-angle X-ray scattering and tryptophan fluorescence studies of phospho­ethano­lamine transferase (EptA) suggest conformational flexibility linked to enzyme activity.

## 1. Introduction   

**Figure 1:**
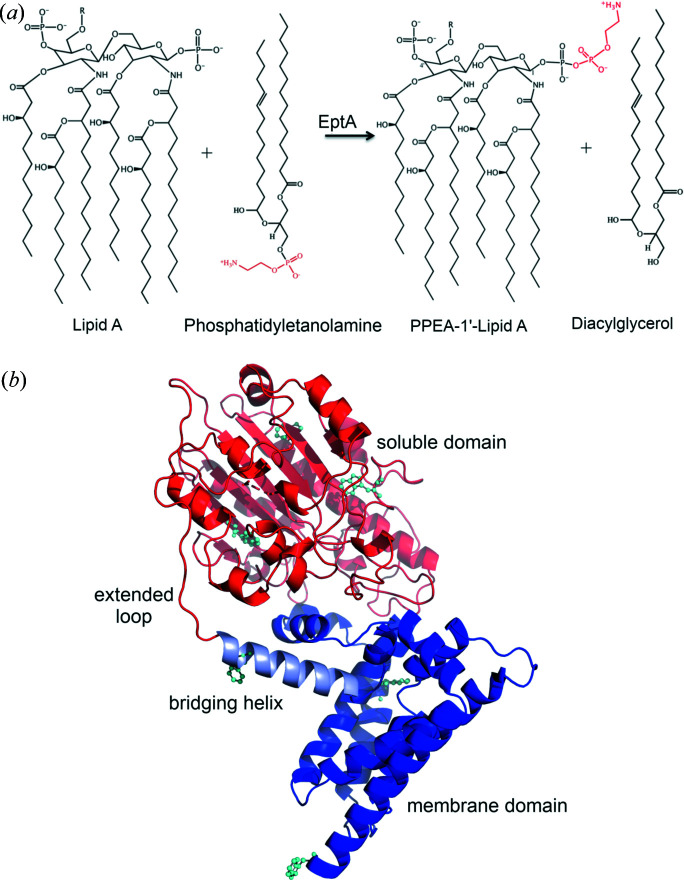
Catalytic reaction and structure of EptA. (*a*) Reaction catalysed by EptA. (*b*) Secondary structure representation of EptA from *N. meningitidis* (PDB entry 5fgn). The N-terminal transmembrane domain is represented in dark blue and the C-terminal soluble domain is represented in red. The bridging helix that connects the transmembrane domain and soluble domain is represented in pale blue. The tryptophan residues in the protein are depicted as cyan balls and sticks.

Pathogenic Gram-negative bacteria have evolved numerous mechanisms to evade the human immune system and developed widespread resistance to host-derived antimicrobials and traditional antibiotics. One such mechanism is to substitute the negatively charged phosphate lipid A head group with phospho­ethano­lamine (PEA) or 4-amino-arabinose residues. Lipid A phospho­ethano­lamine transferase (EptA) transfers PEA from phosphatidyl­ethano­lamine to the 1 and 4′ positions of lipid A [Fig. 1 ▸(*a*)] (Cox *et al.*, 2003 ▸). This modification makes the bacterial surface less negatively charged (Anaya-López *et al.*, 2013 ▸; Peschel, 2002 ▸) and repels the binding of cationic antimicrobial peptides to the surface thus conferring resistance (John *et al.*, 2012 ▸; Trombley *et al.*, 2015 ▸). EptA is found in a number of clinically relevant Gram-negative bacteria (Huang *et al.*, 2018 ▸) and, given its important bio­logical role in reducing the innate immune response, this enzyme has been a target for the development of potential inhibitors as therapeutic agents to treat multi-drug resistant bacterial infections (Kahler *et al.*, 2018 ▸). Towards this aim, and in order to better understand the molecular mechanism involved in substrate binding and enzyme catalysis, the structure of EptA was solved by X-ray crystallography (Anandan *et al.*, 2017 ▸).

The crystal structure of EptA revealed a protein fold with a transmembrane domain and a periplasmic-facing soluble domain linked by an extensive loop and a bridging helix [Fig. 1 ▸(*b*)] (Anandan *et al.*, 2017 ▸). The structure also revealed a tetrahedrally co-ordinated Zn^2+^ ion close to the catalytic nucleophile, Thr280, and a bound *n*-do­decyl-β-d-maltoside (DDM) molecule between the soluble and transmembrane domains near to the active site.

The ability of the enzyme to cleave PEA from phosphatidyl­ethano­lamine in the absence of lipid A (Anandan *et al.*, 2017 ▸) along with the conformation observed in the crystal structure suggests that the two lipid substrates, varying in size, might sequentially access the active site invoking a ping-pong mechanism of catalysis (Wanty *et al.*, 2013 ▸). The bound DDM molecule in the structure appears to position the soluble domain relatively close to the transmembrane domain, thereby locking the enzyme in a conformation that may mimic the phosphatidyl­ethano­lamine-bound state of EptA. In this conformation the binding site would be insufficient in size to accommodate a large lipid A substrate. This further suggests that the enzyme may adopt an alternate conformation to perform the transfer of phospho­ethano­lamine from the active site Thr280 to the lipid A headgroup. Towards establishing the ability of EptA to adopt different conformational poses, the enzyme was also solubilized and purified in *n*-do­decyl-phospho­choline (DPC) detergent micelles, differing from DDM in both structural and ionic properties. Such an approach has enabled studies of the conformational flexibility of other membrane proteins, such as PhoPQ-activated gene P (PagP) in lauroyl di­methyl­amine-*N*-oxide (LDAO) micelles and DPC micelles (Hwang *et al.*, 2004 ▸).

DPC is a zwitterionic detergent containing a phospho­choline head group, highly prevalent among phospho­lipids of membranes, and a do­decyl alkyl tail. Although DPC has gained a reputation for being a harsh detergent, destabilizing and distorting protein structures (Chipot *et al.*, 2018 ▸), it has also provided structural insights into flexible protein systems such as the phosphate transporter Pho89 (Sengottaiyan *et al.*, 2013 ▸) and potassium channel KcsA (Imai *et al.*, 2012 ▸). The effect of DPC on a protein is highly dependent on the specific structure and fold of the macromolecule. Hence, assessment of the protein structural integrity in DPC micelles is essential before undertaking any conformational investigation and interpretations of such studies.

EptA maintains its secondary and tertiary structural integrity in both DDM and DPC micelles as shown by circular dichroism and thermal shift assays, respectively (Anandan *et al.*, 2017 ▸). Limited proteolysis and intrinsic fluorescence experiments performed with EptA in DDM micelles and in DPC micelles suggested that the enzyme adopts different conformations in different detergent micelles (Anandan *et al.*, 2017 ▸). Furthermore, *in silico* molecular dynamics simulations of the enzyme in a bilayer containing dipalmitoyl-*sn*-*glycero*-3-phospho­ethano­lamine lipids indicated that the soluble domain could adopt an alternate position relative to the transmembrane domain (Anandan *et al.*, 2017 ▸). In this simulation the soluble domain was observed to ‘roll over’ the membrane surface yielding an elongated conformation distinct from that observed in the crystal structure. The molecular dynamics study provided impetus to study the conformational flexibility of the enzyme using different experimental biophysical methods.

Structural investigation of EptA in DPC micelles by crystallography has been challenging so far due to the lack of crystal formation. Small-angle X-ray scattering (SAXS) is a powerful technique, particularly useful for conformationally flexible systems (Receveur-Brechot & Durand, 2012 ▸) and especially when partial structural information is available. Furthermore, coupling SAXS to an inline separation technique such as size-exclusion chromatography (SEC) has greatly improved the utility of the measurement for complex systems such as protein:detergent complexes (PDCs) (Mertens & Svergun, 2010 ▸). The method allows separation of the PDC from free detergent micelles, thus providing scattering information of the solubilized system components (Ryan *et al.*, 2018 ▸). Advances in *ab initio* and rigid body modelling methods combined with accurate computation of scattering curves from atomic coordinates can provide reliable models to gain insight into the structural conformation of proteins in solution (Koutsioubas, 2017 ▸; Koutsioubas *et al.*, 2013 ▸). Importantly, these modelling approaches can be used to characterize the structure and function of proteins with significant flexibility, where information on alternate states of the system are difficult or even impossible to obtain by higher resolution methods. The snapshot conformational state captured in the structural models obtained by crystallography can be used in hybrid approaches to further probe conformations of the molecule using data from SAXS and other methods. In this study, we investigate the conformational states of EptA in DDM micelles and DPC micelles using SEC-SAXS and intrinsic fluorescence quenching. Intrinsic fluorescence quenching experiments on mutants of the enzyme where tryptophan residues have been replaced allow us to further probe the local environment of both the soluble and transmembrane domains of EptA as a function of the detergent environment.

The SEC-SAXS study successfully deconvolutes the scattering data of the EptA detergent complex, models the detergent corona around the protein using two independent methods and compares the generated models in different detergent environments. The fluorescence study identifies specific structural regions of the enzyme responsible for conformational changes which localize the interdomain flexibility to the bridging helix linking the soluble and transmembrane domains, and suggests that this flexibility plays a critical role in the catalytic function of the enzyme. This multifaceted biophysical approach not only provides unique information regarding the conformational flexibility of EptA, but also facilitates procedures to study other membrane proteins aimed at understanding the role of conformational flexibility for function.

## 2. Methods   

### 2.1. Plasmid construction for EptA tryptophan mutants   

EptA tryptophan mutants were made by two rounds of splicing by overlap extension polymerase chain reaction (SOE PCR) where the tryptophan residues at positions 126, 148 and 207 were replaced with phenyl­alanines. In the first round of SOE PCR, separate fragments with the W126F and W207F mutations were amplified from wild type *eptA* using primers containing these mutations. In the second round of SOE PCR, these fragments were used as a template by which overlapping complementary ends allowed for annealing and formation of the entire gene, ‘sewing’ the fragments together to create a single mutant construct (W126F) and a double mutant construct (W126F/W207F). This two-step process was re­peated to introduce the W148F mutation, using the W126F mutant as the template to make the double mutant (W126F/W148F) and using the W126F/W207F double mutant as the template to make the triple mutant (W126F/W207F/W148F). Table S1 of the supporting information lists the primers used during each round of SOE PCR. The mutants were cloned into the NcoI and BamHI sites of the pET28a (+) vector. Each constructed mutant plasmid was verified by colony PCR and restriction endonuclease digest as well as DNA sequencing.

For expression in *Neisseria meningitidis* cells, the mutants were excized from the respective pET28a (+) vectors using the restriction enzymes XbaI and SalI and cloned into the XbaI and SaI sites of a neisserial shuttle vector. This vector consists of the gonococcal cryptic fragment from pYT250 (Tzeng *et al.*, 2002 ▸) ligated into the low copy plasmid pHSG576 containing a chloramphenicol resistance marker and two different origins of replication (Takeshita *et al.*, 1987 ▸), enabling replication in both *Escherichia coli* and *Neisseria* species. It also contains an *ompR* promoter to drive downstream gene expression and a synthetically produced multiple cloning site. Successful cloning of both of mutants was confirmed by colony PCR and restriction endonuclease digest.

### 2.2. Polymyxin B sensitivity assay   

Polymyxin B sensitivity was determined via E-strip testing. Following natural transformation (Janik *et al.*, 1976 ▸) of the shuttle vectors into strain NMBΔ*eptA*, overnight growth on gonococcal base agar supplemented with 0.4%(*w*/*v*) d-glucose solution and 0.68 m*M* iron(III) nitrate (GCGF) of each strain was collected using a sterile loop and inoculated in 5 ml of GC broth [1.5%(*w*/*v*) special peptone, 0.4% dipotassium phosphate, 0.1% potassium di­hydrogen phosphate and 0.5% sodium chloride]. The broth suspensions were centrifuged at 60*g* for 2 min to remove clumps and standardized to an OD_560_ of 0.4. Standardized suspensions were then spread onto GCGF plates in volumes of 100 µl. GCGF plates were allowed to air-dry for 10 min and polymyxin B E-strips (BioMérieux) were then placed in the centre of the plates. GCGF plates were then incubated overnight at 37°C with 5% CO_2_ and the zone of inhibition was reported.

### 2.3. Protein expression and purification   

EptA for SEC-MALS and SEC-SAXS experiments was expressed using the pTrc99A expression vector as detailed in the work by Anandan *et al.* (2017 ▸). The mutants used for the tryptophan fluorescence experiments were expressed using the pET28a (+) expression vector designed as described above. All proteins were purified in DDM micelles and DPC micelles as detailed in the work by Anandan *et al.* (2017 ▸). The vectors containing the WT *eptA* gene with a C-terminal hexahistidine tag were expressed in *E. coli* strain BL21 (DE3) pLysS while the mutants were expressed in *E. coli* BL21 (DE3) Rosetta 2. The cultured *E. coli* cells were harvested by centrifugation (12 290*g* for 1 h at 4°C) and lysed in 50 m*M* phosphate buffer pH 7.5 containing 300 m*M* NaCl and 1 m*M* PMSF using an Emulsiflex C5 high-pressure homogenizer (Avestin Emulsiflex C5). The membranes from the lysate were isolated by ultracentrifugation (185 511*g* for 1 h at 4°C). EptA was solubilized and separated from the membranes by incubating the lysate for 4 h with 1% detergent (DDM or DPC) in 50 m*M* phosphate buffer pH 7.5 containing 300 m*M* NaCl, 10 m*M* imidazole and 1 m*M* PMSF followed by ultracentrifugation (185 511*g* for 1 h at 4°C).

Metal-chelating chromatography and size-exclusion chromatography were used for EptA purification. The supernatant obtained from ultracentrifugation was applied to a pre-equilibrated HisTrap crude extract chelating affinity column (GE Healthcare) with the binding buffer [50 m*M* sodium phosphate pH 7.5, 300 m*M* sodium chloride, 20 m*M* imidazole and 3× CMC detergent (0.023% DDM or 0.14% DPC)]. The bound protein was then eluted with the elution buffer [50 m*M* sodium phosphate pH 7.5, 300 m*M* sodium chloride, 500 m*M* imidazole and 3× CMC detergent (0.023% DDM or 0.14% DPC)] using an ÅKTApurifier FPLC system (GE Healthcare). The peak corresponding to EptA was pooled, buffer exchanged into 50 m*M* sodium phosphate pH 7.5, 150 m*M* sodium chloride and 3× CMC detergent (0.023% DDM or 0.14% DPC) using a PD-10 desalting column (GE Healthcare) and applied to a size-exclusion chromatography column (Superdex 200 10/30, GE Healthcare) equilibrated with 50 m*M* HEPES pH 7.0, 100 m*M* NaCl and 3× CMC detergent (0.023% DDM or 0.14% DPC). Fractions of EptA from the elution peak were concentrated using centrifugal filter units (Vivaspin 20 MWCO 100 000, GE Healthcare for DDM purified EptA and Vivaspin 20 MWCO 50 000, GE Healthcare for DPC purified EptA) to 7 or 2 mg ml^−1^ for protein used in SEC-SAXS work and in fluorescence work, respectively, as determined by the absorbance at 280 nm and calculated molar extinction coefficient (73 980 *M* ^−1^ cm^−1^ for WT EptA, 62 980 *M* ^−1^ cm^−1^ for the double mutant and 57 480 *M* ^−1^ cm^−1^ for the triple mutant).

### 2.4. Circular dichroism spectropolarimetry   

The folded state of WT and mutant EptA was confirmed following buffer exchange into 20 m*M* sodium phosphate pH 7.0, 3× CMC detergent (0.023% DDM or 0.14% DPC) with a 5 ml HisTrap Desalting Column using an ÅKTApurifier FPLC system and diluted to a concentration of 0.1 mg ml^−1^. The far- UV spectral region was measured at 20°C on a circular dichroism (CD) spectrophotometer (Jasco J-720) using a 1 mm path length quartz cuvette. Data were collected every 1 nm in the 190–260 nm wavelength region with an integration time of 1 s per step and three accumulations. The CD spectra were collected in triplicate, corrected for the buffer baseline and the signal-to-noise ratio was improved using a Savitzky–Golay filter with the minimum convolution width (of five data points) using *Spectra Analysis* software (version 1.53.07 for Windows 95/NT, Jasco Corp.).

### 2.5. SEC-MALS data collection and analysis   

SEC-MALS analysis of EptA in DDM and DPC micelles was performed using a 10/300GL Superdex 200 column (GE Healthcare) coupled to a Viscotek TDA tetra detector array (Malvern). The SEC-MALS system was equilibrated with 50 m*M* HEPES pH 7.0, 100 m*M* NaCl and 3× CMC detergent (0.023% DDM or 0.14% DPC) to obtain a flat baseline for all the detectors. The instrument was then calibrated against 100 µl of 1 mg ml^−1^ bovine serum albumin prepared in 50 m*M* HEPES pH 7.0, 100 m*M* NaCl and 3× CMC of respective detergent (0.023% DDM or 0.14% DPC). The integrity of EptA purified in DDM micelles and DPC micelles was analysed by injecting 100 µl of the protein at a concentration of 1 mg ml^−1^ into the SEC-MALS system and flowing the sample at 0.3 ml min^−1^. Viscotek’s *OmniSEC* software was used for data collection and analysis. The multi-detector copolymer method was used for analysis and molecular weight determination of protein:detergent complex (PDC) and micelles eluted from the column (Pollock *et al.*, 2012 ▸). Peak limits for PDC and detergent micelles were manually defined and the d*n*/d*c* values for protein (0.185 ml g^−1^) and detergents (0.1608 ml g^−1^ for DDM and 0.1398 ml g^−1^ for DPC) were used for the molecular mass calculations.

### 2.6. SEC-SAXS data collection and analysis   

Inline SEC-SAXS was performed at the SAXS/WAXS beamline at the Australian Synchrotron. Protein samples were subjected to size-exclusion chromatography using a S200 10/300 increase column (GE Healthcare) attached to a Shimadzu HPLC system and passing through a capillary within a SAXS coflow cell (Kirby *et al.*, 2016 ▸; Ryan *et al.*, 2018 ▸). The column was pre-equilibrated with 50 m*M* HEPES pH 7.0, 100 m*M* sodium chloride, 0.1% sodium azide and appropriate detergent concentration (4× CMC of DDM or 3× CMC of DPC). 50 µl of 7.0 mg ml^−1^ EptA purified in DDM micelles or 7.0 mg ml^−1^ EptA purified in DPC micelles was injected onto the column and eluted at 0.2 and 0.4 ml min^−1^, respectively. SAXS data were acquired using X-rays at an energy of 12 kEV and a flux of 3.1 × 10^12^ photons s^−1^. As data were collected on two different occasions, EptA in DPC SAXS data were collected on a Dectris PILATUS 1M detector at 2.5 m (providing the *q* range 0.005–0.3 Å) and EptA in DDM SAXS data were collected using a Dectris PILATUS 2M detector at 2 m (providing the *q* range 0.01–0.5 Å). The frames were collected continuously and integrated every 5 s. The SAXS data collection and data reduction statistics as well as details of the software used for data analyses are given in Table S2. The program *SCATTERBRAIN* 2.82, developed at the Australian Synchrotron, was used to reduce the two-dimensional scattering patterns to one-dimensional profiles followed by the *ATSAS* 3.0 software suite for post-experiment data analysis (Franke *et al.*, 2017 ▸). The integrated errors from the output of *SCATTERBRAIN* were of two standard errors and had to be divided by two to meet the standard definition of χ^2^ to accurately reflect the standard of the fit (Ryan *et al.*, 2018 ▸). *CHROMIXS* (Panjkovich & Svergun, 2018 ▸) was then used to subtract buffer frames (frames ∼100–155) from the PDC elution peak frames to generate the SAXS curve for further analysis. The radius of gyration (*R*_g_) was measured for frames across the protein elution peak, the traces of which were derived from the SAXS/WAXS pipeline, which is based on *AUTORG*, but is more sensitive to noise in the low *q* range. Frames from 391 to 409 for EptA in DDM micelles and frames from 364 to 389 for EptA in DPC micelles were selected for analysis. *PRIMUS* (Konarev *et al.*, 2003 ▸) was used for plotting and inspecting the *CHROMIXS*-generated SAXS curve, *AUTORG* was used for generating *I*(0) and *R*_g_, *DATGNOM* was used to generate the *P*(*r*) functions (Franke *et al.*, 2017 ▸), and *DATCMP* (Franke *et al.*, 2017 ▸; Petoukhov *et al.*, 2012 ▸) was used to compare the similarity of scattering curves. The dimensionless Kratky plot was calculated according to the work by Durand *et al.* (2010 ▸) using the value determined by Guinier analysis, and *P*(*r*) functions were computed from the scattering curves by an indirect transform method in *GNOM* (Svergun, 1992 ▸). The molecular weight was calculated from *I*_0_ using the method by Mylonas & Svergun (2007 ▸), with a partial specific volume calculated using the weight average composition of the PDC from the *MALS* data, a partial specific volume for EptA calculated from the sequence using *MulCH* (Whitten *et al.*, 2008 ▸), and the quoted partial specific volumes for the DDM and DPC from *Anatrace* (https://www.anatrace.com). The electron density of the solvent and PDC was calculated from the composition and elemental makeup. The concentration of the PDC was calculated from the in-line UV absorbance measurements, and an A280 nm extinction coefficient for the PDC was calculated using *PROTPARAM*.

### 2.7. Model building   

#### 2.7.1. *MEMPROT* and *DADIMODO*   

*MEMPROT* (release 2.2) (Perez & Koutsioubas, 2015 ▸) was used to generate hybrid models of the protein:detergent complexes, with the high-resolution structure of EptA (PDB entry 5fgn; Anandan *et al.*, 2017 ▸) used to model the protein component and the detergent corona (DDM or DPC) encircling the transmembrane region of EptA modelled as dummy atoms. The crystal structure lacked the first eight residues at the N terminus and the hexahistidine tag at the C-terminus. The crystal structure lacking the above-mentioned residues was oriented semi-manually using the *PyMOL* software following the instructions from the *MEMPROT* package such that the transmembrane domain aligned parallel to the *z* axis. The elevation and orientation of the structure with respect to the *xy* plane was adjusted (Perez & Koutsioubas, 2015 ▸) in such a way that the aromatic residues forming the aromatic belt of EptA (Anandan *et al.*, 2017 ▸) aligned perpendicular to the *z* axis. *MEMPROT* generated models of the detergent corona around the EptA structure were made by sampling five geometrical parameters, namely ‘*a*’ (height of the detergent torus), ‘*b*’ [cross section of the torus with major (*b.e*) and minor (*b*/*e*) axes], ‘*t*’ (thickness of the hydro­philic shell), ‘*e*’ (ellipticity) and ‘ω’ (the angle of rotation of the corona around the *z* axis) to fit the experimental SAXS curve. The inner hydro­phobic and outer hydro­philic regions of the corona were defined by factoring in the electron densities of the corresponding detergents. Electron density used for modelling the DDM corona was 0.277 e Å^−3^ for the hydro­phobic portion and 0.520 e Å^−3^ for hydro­philic portion of the DDM molecules, whereas the electron density for modelling DPC corona was 0.277 e Å^−3^ for the hydro­phobic portion and 0.490 e Å^−3^ for the hydro­philic portion (Lipfert *et al.*, 2007 ▸). The program calculated parameter values to generate models that best fitted the experimental curve. The agreement between the model generated and the SAXS experimental data is assessed by the χ^2^ value generated by *CRYSOL* (Svergun *et al.*, 1995 ▸) (Table 1 ▸).

To improve the modelling guided by the SAXS data and to optimize the relative positioning of the soluble and transmembrane domains of EptA, the program *DADIMODO* (Evrard *et al.*, 2011 ▸) was used. The *MEMPROT* generated model was used as a starting structure for the *DADIMODO* genetic algorithm. Both the soluble and transmembrane domain structures were kept rigid while the region linking both the domains, namely the bridging helix and the extended loop region, were defined as flexible regions for the program.

#### 2.7.2. *MPBUILDER* and *CORAL*   

All-atom models of the protein:detergent complexes were generated and refined against the SAXS data using *MPBUILDER* (Molodenskiy *et al.*, 2021 ▸) coupled with the rigid-body modelling program *CORAL* (Petoukhov *et al.*, 2012 ▸). As the crystal structure lacked the first eight residues and the histidine tag, these were incorporated into the initial model prior to further modelling. An initial approximation of the number of bound detergent molecules was made based upon the SEC-MALS analysis, and 160 DPC or 225 DDM monomers were added to the transmembrane domain of EptA (PDB entry 5fgn) using the *PACKMOL* routine (Martínez *et al.*, 2009 ▸) implemented in *MPBUILDER*. The transmembrane domain was pre-oriented for membrane insertion using the PPM server (Lomize *et al.*, 2012 ▸) located at the OPM database website (http://opm.phar.umich.edu). The soluble and detergent-bound transmembrane domains were used as input rigid bodies in *CORAL*, and the position of the soluble domain relative to the transmembrane domain refined against the SAXS data, with the linker region between the domains (34 residues) and terminal residues defined as flexible dummy residues.

#### 2.7.3. Flexibility analysis by the *Ensemble Optimization Method*   

Using the low-angle range (*q* < 0.1 Å) of the SAXS data, corresponding to the overall shape of the protein:detergent complexes only, the *Ensemble Optimization Method* (*EOM*) (Tria *et al.*, 2015 ▸) was applied to obtain an estimate of the intrinsic flexibility of the EptA protein solubilized in DDM or DPC detergents. The same rigid bodies as described for the *MPBUILDER*/*CORAL* modelling were used as input for the generation of a pool of 10 000 models for each detergent type, with the inter-domain linker and termini defined as flexible and the soluble domain allowed to sample all possible conformations. Conformations generated that formed steric clashes with the detergent shell (van der Waals cut-off < 0.4 A) were discarded, leaving approximately 5000 conformations for the final pool and subsequent analysis for both DDM and DPC EptA complexes. Scattering intensities for each conformer in each pool were computed using *CRYSOL* (Svergun *et al.*, 1995 ▸). The intensities corresponding to the pool were then subjected to the genetic algorithm routine *GAJOE* within *EOM*, and a subset of conformations that best fit the experimental data were selected through a series of random selection and elitism steps. The flexibility assessment was then performed on size distributions corresponding to the radii of gyration of the corresponding conformers selected and compared with that of the pool. The statistic *R*_flex_ (Tria *et al.*, 2015 ▸) was calculated for both the selected ensemble and the pool, where *R*_flex_ approaching 100% indicates a fully flexible system of random conformers.

### 2.8. Intrinsic tryptophan fluorescence measurements   

Intrinsic fluorescence was monitored using an EnSpire Multimode plate-reader (Perkin Elmer). Sample excitation was carried out at 295 nm (100 flashes; <8 nm bandwidth) in order to selectively excite tryptophan residues and emission spectra were measured from 315 to 405 nm at 1 nm intervals. Protein samples at 12 µ*M* concentration in 50 m*M* HEPES pH 7.0, 50 m*M* NaCl and 3× CMC detergent (0.023% DDM or 0.14% DPC) to a final volume of 100 µl were gradually quenched by the addition of small aliquots (1 or 2 µl) of 4 *M* potassium iodide also containing 0.2 m*M* sodium thio­sulfate to prevent the formation of triiodide (I_3_^−^). Non-quenched samples were maintained at the same ionic strength by including equivalent volumes of potassium chloride. For the intrinsic fluorescence of denatured protein, samples also contained 4 *M* guanidine hydro­chloride as a denaturant and 0.2 *M* β-mercapto­ethanol to maintain reducing conditions throughout the experiment. All samples were incubated for 15 min at 20°C in a UV-Star half-area microplate (Greiner Bio-One). Spectra were recorded from three replicate protein wells and corrected by subtracting measurements from three replicate protein-free wells of the same volume and equivalent salt and buffer compositions.

Data obtained from quenching experiments were fitted to equation (1) in order to determine the Stern–Volmer constant (*K*_SV_), where [I^−^] corresponds to the iodide concentration:
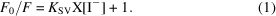
The relative fluorescence intensity of unquenched to quenched protein (*F*_0_/*F*) was obtained from triplicate spectra by dividing the maximum protein fluorescence emission in the presence of KCl by the maximum protein fluorescence emission with an equivalent concentration of KI. This corrects for changes to the ionic strength and volume of samples with each addition of the quenching or non-quenching salt (*i.e.* KI and KCl, respectively). Equation (1) was fitted to the linear regions of the quenching data using linear regression in the *GraphPad Prism* software (version 5.02 for Windows, *GraphPad* Software Inc.).

## 3. Results   

### 3.1. Characterization of the protein:detergent complex by SEC-MALS analysis   

EptA purified in DDM micelles and DPC micelles were analysed by SEC-MALS in order to establish that the protein was monodisperse in different detergent micelles and amenable to SAXS studies. Figs. S1(*a*) and S1(*b*) of the supporting information display the overlayed chromatograms obtained from the UV absorption at 280 nm and refractive index (RI) signal superimposed on the right-angle scattering signal for EptA in DDM micelles and DPC micelles, respectively. EptA in DDM micelles eluted at 14.11 ml as a single peak as detected by the UV absorbance while the RI signal displayed an additional peak at 15.9 ml corresponding to free DDM micelles. A curvature across the elution peak was evident for EptA in DDM micelles suggesting a possible dynamic equilibrium or concentration-dependent association. While preliminary SEC-SAXS experiments altering protein/detergent concentration ratios were carried out as an optimization process (data not presented) the current ratio of [EptA]:[DDM] of 7 mg ml^−1^:4× CMC was chosen for the final SEC-SAXS analysis as the sample in this condition behaves more uniformly and mondisperse. EptA in DPC micelles eluted at 14.41 ml and the free DPC micelles eluted at 16.74 ml. Although a small overlap between the PDC peak and detergent micelle peak was observed for both samples, the peak boundaries were carefully set to minimize the overlap before data analysis was undertaken. The average molecular weight (MWt) of the EptA:DDM complex estimated by RI was 160.3 ± 8.0 kDa and the calculated MWt of EptA was 58.31 kDa. For the EptA:DPC complex, the average MWt was estimated to be 109.0 ± 3.4 kDa and that of EptA alone was estimated at 62.85 kDa. The molecular weight estimation of EptA in DDM micelles was slightly lower than the theoretical value, estimated from *PROTPARAM* (Gasteiger *et al.*, 2005 ▸) (61.40 kDa) while the estimation of EptA in DPC micelles was closer to the theoretical value. Based on these results the average numbers of DDM and DPC molecules associated with the protein:detergent complex were estimated to be 200 and 123, respectively.

### 3.2. Conformational analyses of the protein:detergent complexes by SEC-SAXS   

The conformation of EptA purified in the two aforementioned detergent micelles was analysed by SEC-SAXS at the Australian Synchrotron. For both detergent systems, the elution profile from the S200 10/300 increase SEC column revealed a PDC peak slightly overlapped with the detergent micelle peak; however, the separation was sufficient to provide unambiguous SAXS signals from the PDC and detergent micelle species as shown by the similarity of the extracted SAXS parameters across the peak maxima (Fig. S2 of the supporting information). The molecular weight for the PDC from the SAXS signal was determined as described by Mylonas & Svergun (2007 ▸), where the partial specific volume was estimated from the MALS data, and the protein concentration was determined using inline A280 nm absorbance measurements, and the extinction coefficient was obtained from the EptA sequence using *PROTPARAM* (Gasteiger *et al.*, 2005 ▸). Additionally, a comparable result was obtained through a Bayesian approach for molecular weight determination, carried out using the *ATSAS* software (Franke *et al.*, 2017 ▸; Hajizadeh *et al.*, 2018 ▸). Molecular weight analyses of the PDC species for frames selected from the SEC-SAXS peak were provided by the Bayesian molecular weight calculation in *ATSAS*. The values ranged from 200 to 218.5 kDa for the EptA:DDM complex and from 125.5 to 136.37 kDa for the EptA:DPC complex [Fig. S2(*a*)]. Guinier analyses of the PDC peak for the sample in DPC micelles exhibited less variation in *R*_g_ across the peak than was observed for the sample in DDM micelles [Fig. S2(*b*)]. While these values for molecular weight differ somewhat from those determined by the SEC-MALS analyses, other examples show deviations of 10–20% (Gimpl *et al.*, 2016 ▸; Mirandela *et al.*, 2018 ▸) and are well within the accuracy expected from SAXS determination of molecular weight (Mylonas & Svergun, 2007 ▸). Additionally, the differences in the concentrations of the protein and detergent within each experiment may account for the variation observed in the number of detergent molecules by SEC-MALS and by SEC-SAXS.

**Figure 2:**
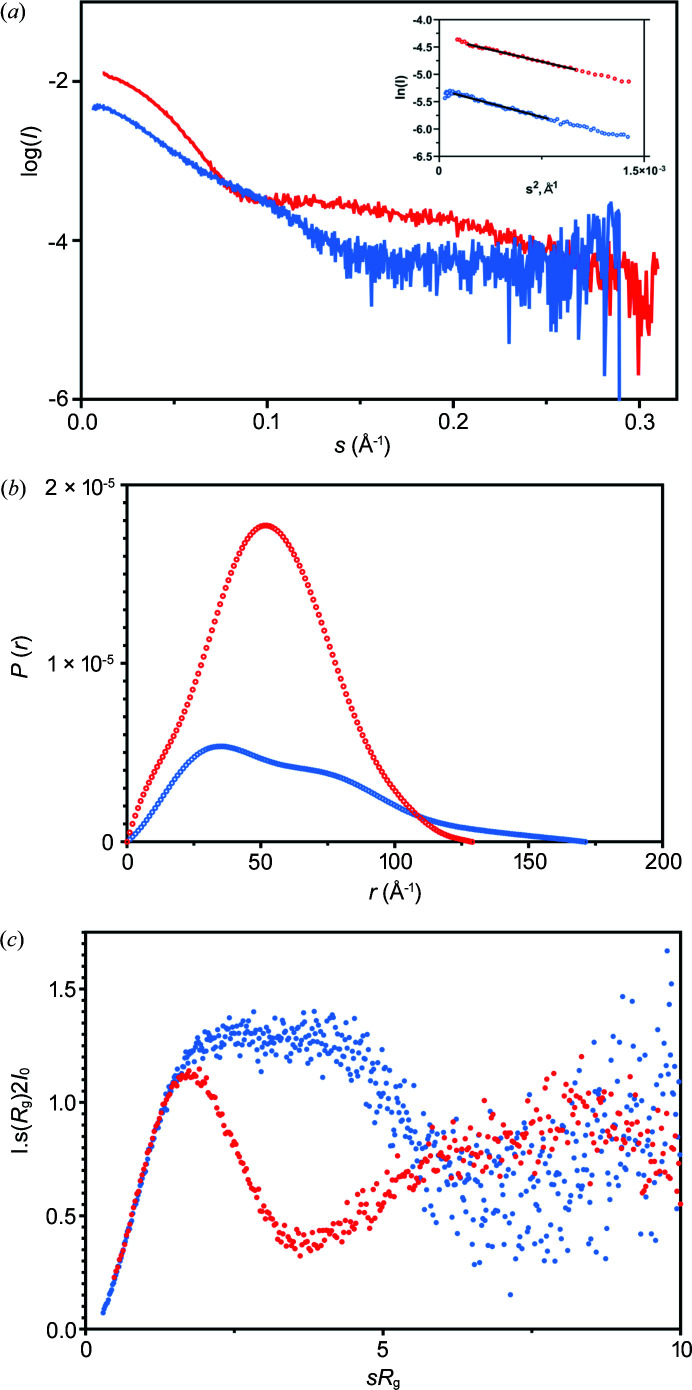
SEC-SAXS results for the EptA:DDM complex and EptA:DPC complex. (*a*) *I*(*s*) versus *s*, as log–linear plots with the inset showing the Guinier fits (black line) laid on the experimental data (open symbols). (*b*) Profiles for the distance distribution function, *P*(*r*), in arbitrary units versus *r* in ångströms for the data shown in (*a*), normalized for ease of comparison. (*c*) Dimensionless Kratky plots for the data in (*a*).

The averaged, buffer-subtracted SAXS profile of the EptA:DDM complex and the EptA:DPC complex are presented in Fig. 2 ▸(*a*). Guinier analysis of both the EptA:DDM complex and the EptA:DPC complex revealed linear Guinier plots [Fig. 2 ▸(*a*) inset] without evidence of aggregation/interparticle interaction in the samples. For the EptA:DDM complex an *R*_g_ value of 42.0 ± 0.7 Å was determined and the distance distribution function, *P*(*r*), showed a single bell-shaped distribution with a maximal dimension (*D*_max_) of 129 Å, suggesting a compact globular structure. For the EptA:DPC complex, an *R*_g_ value of 45.8 ± 0.4 Å was determined. The *P*(*r*) showed a peak with an extended tail, characteristic of an elongated particle shape, and a *D*_max_ of 171 Å. The extracted SAXS parameters and the significant differences in the shape of the distance distributions clearly show the EptA:DPC complex to be larger and more extended than the EptA:DDM complex (Kikhney & Svergun, 2015 ▸). The symmetric bell-shaped *P*(*r*) distribution of the EptA:DDM complex and extended *P*(*r*) distribution for the EptA:DPC complex are shown in Fig. 2 ▸(*b*).

The dimensionless Kratky plot (assessing the globularity and flexibility of the protein) shows notable differences in the intermediate *s* range for EptA in different detergent systems [Fig. 2 ▸(*c*)]. The EptA:DDM complex exhibits a bell-shaped curve, with maximum of 1.147 at 1.9 *sR*_g_ indicating a folded/globular shaped molecule with limited flexibility. The EptA:DPC complex shows a maximum of 1.4 at 2.8 *sR*_g_ with an additional shoulder at the intermediate *s* region and converging at higher angles, suggesting that this complex is both more elongated and potentially more flexible than the EptA:DDM complex (Durand *et al.*, 2010 ▸; Rambo & Tainer, 2011 ▸).

### 3.3. *MEMPROT* and *DADIMODO* modelling results   

The SAXS profile of a PDC comprises signal from both the detergent solubilized protein and from the protective amphiphilic corona formed by detergent molecules. In order to gain insight into the conformation adopted by the protein in different detergent environments, models of the PDC consistent with the experimental SAXS curves were generated (Table 1 ▸).

**Figure 3:**
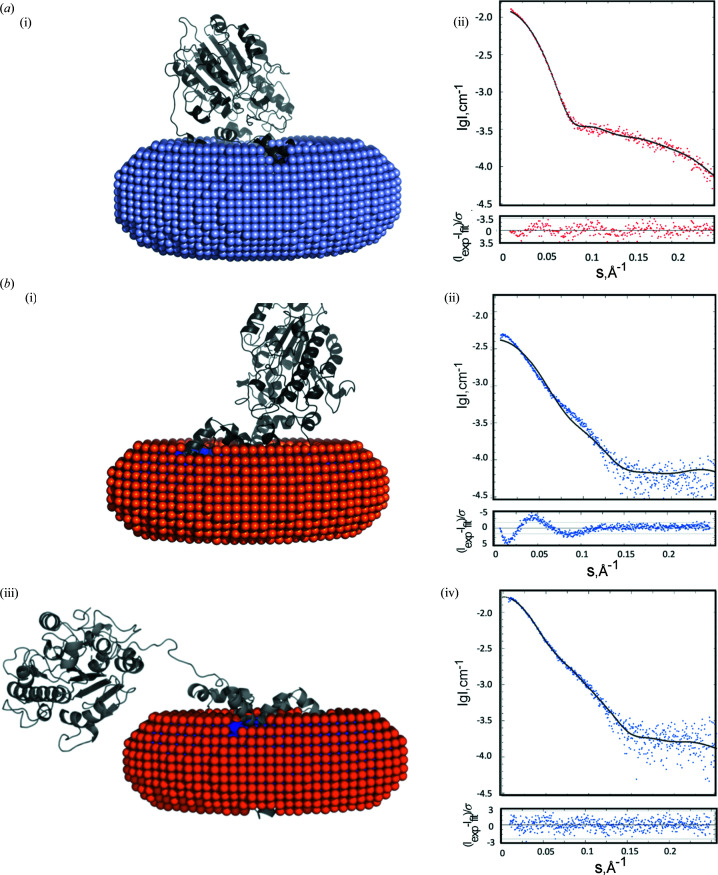
*MEMPROT* and *DADIMODO* modelling results. (*a*) Model and scattering fit for the EptA:DDM complex and (*b*) the EptA:DPC complex. (*a*) (i) Representative *MEMPROT* generated model for the EptA:DDM complex. The atomic structure of EptA is represented in cartoon mode and coloured grey. The DDM corona around EptA is represented as pale blue spheres. (*a*) (ii) Comparison of the SAXS experimental result (red dots) with the *MEMPROT* calculated scattering curve (black solid line). The lower inset shows the error-weighted residual difference plot for the experimental SAXS model and the *MEMPROT* generated model (red dots). (*b*) (i) Representative *MEMPROT* generated model for the EptA:DPC complex. The atomic structure of EptA is represented in cartoon mode and coloured grey. The DPC corona around EptA is represented as orange spheres. (*b*) (ii) Comparison of the SAXS experimental result (blue dots) with the *MEMPROT* calculated scattering curve (black solid line). The lower inset shows the error-weighted residual difference plot for the experimental SAXS data and the *MEMPROT* generated model (blue dots). (*b*) (iii) Model of the EptA:DPC complex obtained with *DADIMODO*, assigning the soluble domain and transmembrane domain as rigid bodies connected by a flexible bridging helix and linker region. The atomic structure of EptA is represented in cartoon mode and coloured grey and the DPC corona around EptA is represented as orange spheres. (*b*) (iv) The lower inset shows the error-weighted residual difference plot for the experimental SAXS data and the *DADIMODO* generated model (blue dots).

*MEMPROT* (Pérez & Koutsioubas, 2015 ▸) was employed to model the detergent assemblies as course grained dummy atoms around the hydro­phobic region of the EptA crystal structure (PDB entry 5fgn; Anandan *et al.*, 2017 ▸). For the EptA:DDM complex the best fitting models built an elliptical DDM torus around the EptA molecule [Fig. 3 ▸(*a*) panel (i)] with an inner hydro­phobic region 31 Å-thick and an outer hydro­philic region 4.8 Å-thick [Fig. S3(*a*) panels (ii) and (iii)]. The derived SAXS profile for this model closely matched the experimental SAXS curve [χ^2^ = 1.2; Fig. 3 ▸(*a*) panel (ii)]. *MEMPROT* estimated 235 ± 37 DDM molecules in the detergent corona which corresponded well with the estimate obtained by SEC-MALS (200 DDM molecules).

For the PDC in DPC the *MEMPROT* models generated [Fig. 3 ▸(*b*) panel (i)] and their corresponding SAXS profile departed considerably from the experimental curve [χ^2^ > 3.5; Fig. 3 ▸(*b*) panel (ii)]. Based on the primary SAXS analysis discussed above, the possible conformational change to a more extended structure was investigated through further refinement of the *MEMPROT* EptA:DPC complex model using *DADIMODO* (Evrard *et al.*, 2011 ▸). *DADIMODO* uses a genetic algorithm approach to sample domain positions and select the best fitting model conformations using SAXS data. The *MEMPROT* generated model of the EptA:DPC complex resulted in a DPC corona with a hydro­phobic inner region of 24.7 Å and a hydro­philic outer region of 3.7 Å [Fig. S3(*b*) panels (ii) and (iii)]. The number of DPC detergent molecules modelled in the detergent corona was 190 ± 3; however, as the EptA model is constrained to the crystallographic coordinates, if the system is indeed dynamic this estimate may not be reliable and can be taken only as a rough guide. This model of EptA in the DPC torus was used as input to *DADIMODO* with the bridging helix and extended loop (residues 174–230) defined as the flexible region of the structure. *DADIMODO* generated five different models [a representative model is shown in Fig. 3 ▸(*b*) panel (iii)] which generated calculated SAXS profiles that fit well with the experimental SAXS data [χ^2^ = 1.05–1.07; Fig. 3 ▸(*b*) panel (iv)]. All five models were similar, displaying the soluble domain rolled away from the transmembrane domain with an increased distance between the centre of mass of the domains when compared with the crystal structure of EptA.

The results of this analysis provide experimental and structural evidence that the protein in the two different detergent micelle environments adopts different conformations with the orientation of the two protein domains varying in a detergent-dependent manner. Interestingly, the altered conformation adopted by EptA in DPC micelles resembled the *in silico* molecular dynamics simulations of the enzyme in a lipid bilayer (Anandan *et al.*, 2017 ▸) as a more open structure compared with that observed in the DDM micelles.

### 3.4. *MPBuilder* and *CORAL* modelling results   

**Figure 4:**
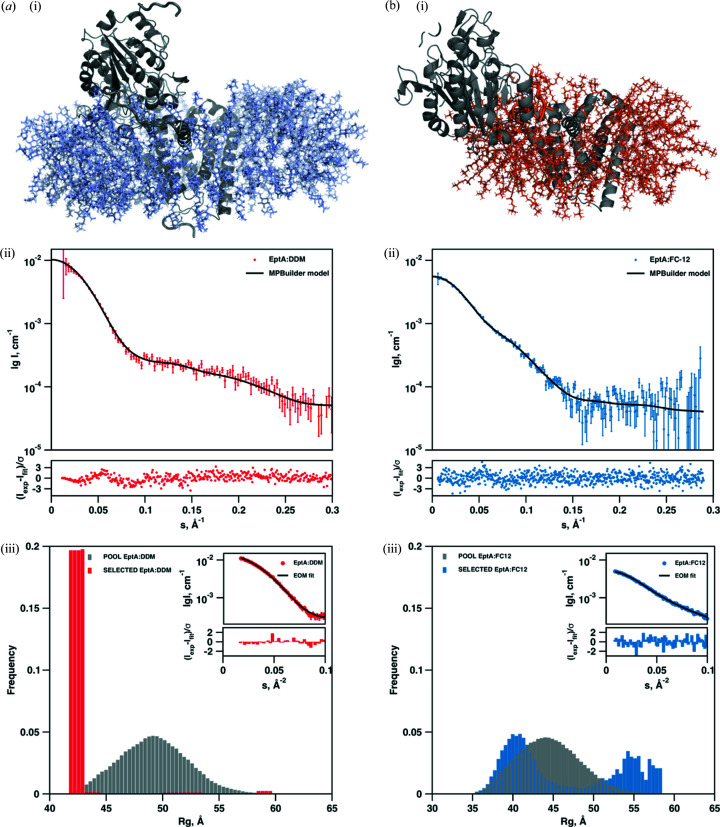
Hybrid SAXS modelling results and flexibility analysis. Results for the (*a*) EptA:DDM complex and (*b*) EptA:DPC complex. (i) Hybrid SAXS models generated for each of the complexes using the combined *MPBuilder*/*PACKMOL*/*CORAL* approach. The atomic structure of EptA is represented in cartoon mode and coloured grey. The detergent corona around EptA is represented as pale blue and orange coloured lines for DDM and DPC, respectively. (ii) Comparison of the SAXS experimental data (coloured red and blue for EptA:DDM and EptA:DPC, respectively) with the scattering curves computed from the best fitting hybrid models (black solid line). The lower insets show the error-weighted residual difference plot for the fits. (iii) Flexibility analysis of the complexes using the *EOM*. Distance profiles (*R*_g_) of the random pools of PDC conformations generated (grey histograms) and of the selected ensembles of conformations that best fit the SAXS data (red and blue histograms for EptA:DDM and EptA:DPC, respectively). The fits of the selected ensembles to the experimental data and residuals are shown in the inset. For EptA:DDM the selected ensemble consists of a narrow population of similar compact conformations, and two wide populations of both compact and extended conformations are selected for EptA:DPC, indicating significant conformational flexibility.

In a parallel approach to the model determination of the EptA complexes with dummy atoms representing detergent, hybrid models were computed using the *MPBuilder* algorithm (Molodenskiy *et al.*, 2021 ▸) combined with the programs *PACKMOL* (Martínez *et al.*, 2009 ▸) and *CORAL* (Petoukhov *et al.*, 2012 ▸). This procedure allowed for the generation of all-atom models of the complexes from the crystal structure of EptA and both DDM and DPC detergent micelles. In addition, this approach modelled the inter-domain loop, allowing for rearrangement of the soluble and transmembrane domains, as well as the terminal residues not observed in the EptA crystal structure. Qualitatively, the models generated for both detergent complexes [Figs. 4 ▸(*a*) and 4(*b*) panel (i)] are similar to those generated from the hybrid *ab initio* approaches employed by *MEMPROT* and *DADIMODO*. The best fitting models for the DDM and DPC complexes again demonstrated different conformations, with a compact structure observed for EptA in the presence of DDM and a significantly more extended structure observed in the presence of DPC.

Though the DPC data were very well described by single models calculated by the hybrid procedure, systematic deviations were observed at higher angles for the data of the protein in DDM micelles [Fig. 4 ▸(*a*) and 4(*b*) panel (ii)]. This led us to speculate that intrinsic flexibility of EptA may differ between the two detergent conditions and that a structural ensemble may better represent the conformation of the protein in solution. *EOM* (Bernadó *et al.*, 2007 ▸) was applied to investigate the flexibility of EptA in DDM and DPC micelles. *EOM* constructs a pool of randomized conformations, using high-resolution structure components as rigid bodies with linkers and terminal regions modelled as flexible dummy residues. From this pool ensembles of conformers are selected through a series of elitism steps, with the final ensemble providing a computed scattering pattern that best fits the experimental data. For both EptA detergent complexes the bridging linker between the soluble and transmembrane domains (including the detergent corona as modelled in the *MPBuilder*/*PACKMOL* protocol) was defined as flexible and a pool of randomized conformations were generated. The selection protocol identified ensembles that best fit the scattering data and the distance distributions of these ensembles compared with that of the initial pool [Fig. 4 ▸(*a*) and 4(*b*) panel (iii)]. For EptA:DDM the distribution of the selected ensemble is narrow and clearly shifted to small *R*_g_ values, indicating that, under these conditions, EptA adopts a small range of compact conformations. The metric of flexibility *R*_flex_ is 34.8%, compared with 89.0% calculated for the pool, indicating that the EptA is not significantly flexible in DDM. In contrast, for EptA:DPC two broad populations of conformations are observed in the distribution at low *R*_g_ and high *R*_g_ values and the calculated *R*_flex_ is 91.6%, compared with 88.6% for the pool. Thus, in DPC EptA exists in a range of conformations and is significantly more flexible. From this analysis the conformational flexibility of EptA was determined to be significantly larger in DPC than in DDM micelles, with the latter again clearly showing a preference for compact structures.

### 3.5. Characterizing the flexible regions of EptA mutants in detergent micelles by intrinsic fluorescence analysis   

Previously, intrinsic tryptophan fluorescence studies were carried out using wild type (WT) EptA, in order to probe conformational states of the enzyme in different detergent micelle environments (Anandan *et al.*, 2017 ▸). While this provided information regarding conformational flexibility of the molecule as a whole it did not give information as to the specific localization of movement with respect to individual domains. EptA contains six tryptophan residues: Trp247, Trp320 and Trp484 localized in the soluble domain, Trp126 and Trp148 in the transmembrane domain, and Trp207 in the bridging helix. In order to probe the conformational states more fully, the tryptophan residues localized in the transmembrane domain and the bridging helix were mutated to phenyl­alanine side chains, leaving only the soluble domain tryptophan residues intact resulting in a triple mutant of the enzyme (EptA_Trp126/148/207Phe_). Additionally, the tryptophans localized only in the transmembrane domain were mutated to phenyl­alanines resulting in a double mutant (EptA_Trp126/148Phe_), leaving the soluble domain tryptophan residues as well as the tryptophan on the bridging helix intact. To ensure that these mutations were not detrimental to enzyme function, the minimum inhibitory concentration (MIC) of each mutant to a model CAMP, polymyxin B, was examined. The *N. meningitidis* NMBΔ*eptA* strain had an MIC of 0.19 µg ml^−1^, and expression of both mutants in this strain could restore polymyxin B resistance to the same level as the WT NMB strain (MIC of 384 µg ml^−1^), indicating the two mutants were fully functional (Table S3).

Each of these mutants was recombinantly expressed, solubilized and purified in both DDM and DPC micelles, and analysed by circular dichroism (CD) spectroscopy to ascertain the folded state of the protein relative to that of the wild-type enzyme under identical detergent conditions (Fig. S4). The double and triple mutants maintained the secondary structure profile observed for the WT enzyme in both detergent micelles, suggesting a conservation of the fold and secondary structure despite the mutations.

**Figure 5:**
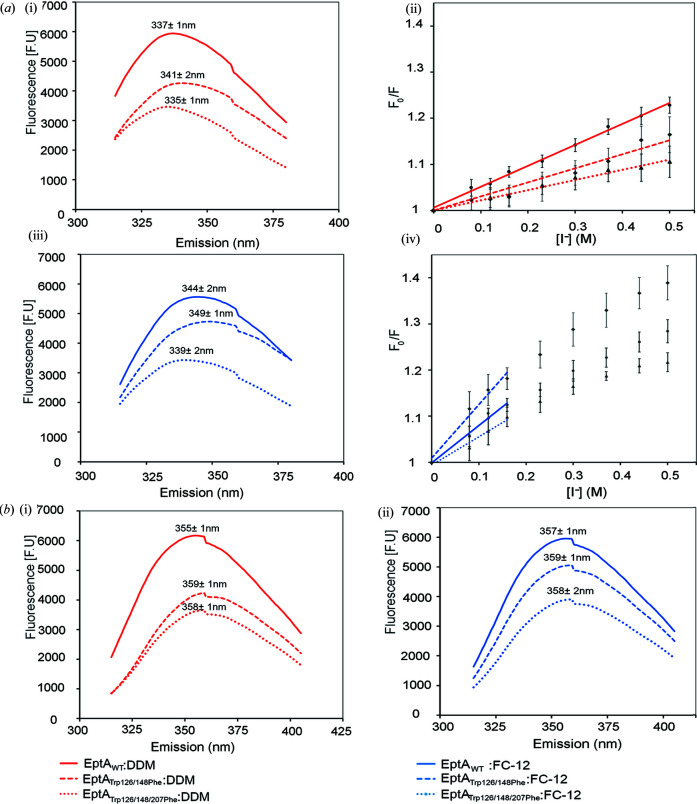
Intrinsic fluorescence spectra and quenching profiles for EptA in different detergent micelles. (*a*) Fluorescence spectra and Stern–Volmer plots for native folded enzyme and (*b*) fluorescence spectra for the denatured enzyme. The emission wavelength maxima (λ_max_) for each condition are indicated above each curve. The red and blue coloured lines represent enzyme in DDM and DPC micelles, respectively. The solid lines represent EptA_WT_, the dashed lines represent EptA_Trp126/148Phe_ and the dotted lines represent EptA_Trp126/148/207Phe_. Panels (i) and (iii) show the emission spectra of the enzymes and panels (ii) and (iv) in (*a*) show the Stern–Volmer plot for quenching using iodide. A linear regression was fitted using *GraphPad* prism with an *R* ^2^ value above 0.95. Where a non-linear relationship was observed, only the initial linear slope was fitted.

Iodide, a hydro­philic quencher, was used to probe the tryptophan environment of EptA solubilized and purified in the different detergent micelles. The intrinsic tryptophan fluorescence emission spectra of EptA in a native folded state and denatured in the presence of 4 *M* guanidinium–HCl and 25 m*M* β-mercapto­ethanol are shown in Fig. 5 ▸(*a*) panels (i) and (iii), and 5(*b*) panels (i) and (ii). As expected, the fluorescence signals from the double and triple mutants were diminished relative to the wild-type enzyme, since they now have four and three tryptophan residues, respectively, whereas the wild-type enzyme has six tryptophan residues.

A comparison of the emission spectra for folded proteins in 0.5 *M* KCl provides insights into the tryptophan microenvironment of each domain, independent of iodide quenching. The triple mutant in DDM micelles was the most blue-shifted sample with a λ_max_ of 335 nm, indicating the burial of tryptophan residues within the hydro­phobic core of the soluble domain [Fig. 5 ▸(*a*) panel (i)]. Solubilization of enzymes in DPC micelles resulted in a red-shift compared with DDM solubilized protein, with the WT, double mutant and triple mutant shifting 7, 8 and 4 nm, respectively [Fig. 5 ▸(*a*) panels (i) and (iii)]. This suggests that EptA in DPC micelles adopt a more open structure with greater overall solvent exposure of the tryptophan residues than protein in DDM micelles. Furthermore, the 8 nm red-shift of the double mutant compared with the 4 nm red-shift of the triple mutant in the two different detergent micelles suggests a more hydro­philic environment in the region of the bridging helix for the protein in DPC micelles as the presence of the tryptophan residue on the bridging helix is the only difference between these mutants. Further support for the role of the bridging helix in opening the structure is evident when comparing each mutant in DPC micelles; the λ_max_ of the triple mutant was 339 nm whereas the double mutant, which still contained the tryptophan residue on the bridging helix, was red-shifted by 10 nm to 349 nm.

In all cases, the denatured samples exhibited a λ_max_ of at least 355 nm, a dramatic red-shift from the native folded protein, demonstrating that the tryptophan side chains are positioned in very hydro­philic environments, consistent with an unfolded protein [Fig. 5 ▸(*b*)]. The λ_max_ of the denatured enzyme was similar in DDM and DPC (355–357 nm for WT EptA, 359 nm for the double mutant and 358 nm for the triple mutant). This consistency indicates that any detergent-driven conformational differences are absent after denaturation.

Fluorescence-quenching experiments with KI support the results obtained from the emission spectra. Quantitative analysis of the effect of quenching was undertaken by analyzing the Stern–Volmer plots for each protein and deriving the Stern–Volmer constants (*K*_SV_) [Fig. 5 ▸(*a*) panels (ii) and (iv), Table 2 ▸]. Quenching studies were not required for denatured protein, as the emission spectra already demonstrated that these proteins had unfolded to the same extent. The Stern–Volmer plots of the WT and mutant proteins in DDM yielded a linear quenching relationship over the entire range of KI concentrations examined. In these cases, a simple collisional mechanism is used to describe the process of tryptophan quenching by iodide. The Stern–Volmer plots corresponding to the WT and mutant proteins in DPC displayed negative curvature which suggests that EptA in the DPC micelle exhibits considerable mobility such that the tryptophan residues often sit at the micelle surface (Hill *et al.*, 1986 ▸). The *K*_SV_ values of folded WT EptA were larger than both mutants in DDM micelles and the triple mutant in DPC micelles [Fig. 5 ▸(*a*) panels (ii) and (iv), Table 2 ▸]. Additionally, *K*_SV_ was larger for WT EptA and for each of the mutants in DPC micelles than those in DDM micelles, suggesting an overall increase in hydro­philicity and quencher accessibility of the tryptophan residues for the protein in DPC micelles. Unlike EptA in DDM micelles, the double mutant exhibited the highest *K*_SV_ value for protein in DPC micelles, demonstrating an increase in solvent exposure of the bridging helix. The location of Trp207 on the bridging helix and the structural conformation of the protein in DPC micelles provides more accessibility of this tryptophan to the KI quencher than the transmembrane domain tryptophan residues (Trp126 and Trp149). Indeed, WT EptA that retained the transmembrane domain tryptophan residues exhibited a lower K_SV_ value in DPC.

## 4. Discussion   

The enzyme chemistry required for lipid A modification by EptA involves the binding of two lipid substrates: phosphatidyl­ethano­lamine as a donor lipid and lipid A as a receiver lipid for the PEA moiety. The enzyme first transfers the PEA moiety from the donor lipid to an active-site threonine (Thr280) to make a PEA-bound enzyme intermediate with the release of di­acyl­glycerol. Lipid A must then bind to the enzyme to enable the transfer of PEA from the enzyme intermediate to make the final modified lipid A product. Previously, our crystallographic structure of EptA, where crystals were grown from recombinant enzyme purified in DDM micelles, showed a closed structure that was able to accommodate a DDM molecule in the binding site (Anandan *et al.*, 2017 ▸). The O3B of this DDM molecule is positioned identically to that of the phosphate oxygen atom covalently bonded to the side chain of the active-site Thr280 in the truncated soluble domain structure of EptA (Wanty *et al.*, 2013 ▸). Further examination of the structure reveals that an enzyme-linked PEA molecule can be accommodated between Thr280 and Glu114, located in the membrane domain. Indeed, this glutamate side chain is well positioned to form a hydrogen bond with the amine of PEA (Anandan *et al.*, 2017 ▸). Based on these observations we hypothesized that the bound DDM molecule mimicked the phosphatidyl­ethano­lamine substrate-bound conformation of the enzyme. However, this conformation would not accommodate the binding of lipid A near to the PEA-bound Thr280 so we presumed that the protein must be able to adopt different conformational states in solution to enable PEA transfer. Molecular dynamic simulations of EptA embedded in the PE/PG system showed that the overall conformation of the enzyme remained close to the starting structure in four of the six simulations, though in two simulations the soluble domain dissociated from the membrane domain and rolled over the membrane surface interacting with the lipid polar heads (Anandan *et al.*, 2017 ▸). This suggested that the protein is capable of adopting different conformations and the current study was undertaken to interrogate the flexibility of EptA by SEC-SAXS and intrinsic fluorescence solution studies. WT EptA and mutant forms of the enzyme, where selective tryptophan residues were replaced with phenyl­alanines, were solubilized and purified in two different detergent systems, namely DDM and DPC, separately.

DDM and DPC detergents contain a 12-carbon acyl chain and a maltoside and phospho­choline head group, respectively. The structural integrity of EptA in these detergent micelles was validated by CD analyses, differential scanning fluorimetry, and limited proteolysis with trypsin and chymotrypsin (Anandan *et al.*, 2017 ▸). The CD spectra of WT protein in both DDM and DPC micelles show a similar profile, suggesting that the secondary structure of EptA is conserved in both detergent micelles. Furthermore, both EptA:DDM and EptA:DPC exhibit distinct melting temperatures suggesting that the enzyme retains a stable tertiary structure in these two detergent systems (Anandan *et al.*, 2017 ▸). EptA:DDM and EptA:DPC resisted both trypsin and chymotrypsin digestion and showed distinct bands up to 12 h further supporting that the protein remains folded in the two detergent systems.

Given that EptA maintains its structural integrity in the detergent micelles used for this study, the results presented here using two different biophysical methods aimed at probing flexibility of the enzyme provide further experimental evidence suggesting that the enzyme is structurally dynamic. For these studies MALS analysis was first performed in order to assess whether the samples were amenable to SAXS studies. However, careful analysis indicated a discrepancy in the molecular weight and thus the MALS data were used mostly to confirm a rough stoichiometry of one protein molecule per micelle, but were not sufficient to confirm the ratio for detergent to protein, most likely due to changes in the concentration between the MALS analysis and the SEC-SAXS analysis. All modelling-based analyses used the molecular weight calculation obtained directly from the SEC-SAXS experiment. The Guinier plot from the SEC-SAXS data showed no evidence of aggregation or unfolding which would have been apparent at low *s*. An increase in flexibility of EPtA in DPC was apparent from the Kratky plots. Further modelling using multiple algorithms indicated that the models obtained were consistent with folded protein, based on the core of the crystal structure, and these models recapitulate the SAXS data closely. Intriguingly, the analyses of the SAXS data using various algorithms have converged to similar models which match well with the results obtained from the MD simulations of EptA in a membrane environment (Anandan *et al.*, 2017 ▸).

Using different tryptophan mutations, the source of the conformational flexibility can be localized to the bridging helix, connecting the soluble domain to the transmembrane domain of the structure. The *K*_SV_ constant obtained from the Stern–Volmer plots reflects the accessibility of the quencher to the tryptophan fluoro­phore and will be smaller with steric hindrance of quencher–fluoro­phore collisions, if the tryptophan residue is located in a buried crevice or within the hydro­phobic protein core (Lakowicz, 2006 ▸). The larger conformational flexibility of EptA in DPC micelles as seen from the Stern–Volmer analysis is consistent with the models determined by *EOM* analysis. In general, the analysis supports the structural observations that the tryptophan residues in the soluble domain are more buried than in the transmembrane domain for protein in each detergent environment since the transmembrane domain tryptophan residues are only present in the WT enzyme. Additionally, the results provide evidence that the conformational flexibility of EptA is localized at the bridging helix and that this flexibility enables the two domains to roll away from one another while the helix becomes more solvent exposed.

A caveat of these studies is that DPC is notoriously well known for causing destabilizing effects in other membrane systems, and thus these results will need further investigation to confirm the exact physiological relevance of the open and closed conformations that we have observed. Nevertheless, the results do provide some insights into the flexible nature of the protein and, intriguingly, align with the results of previous *in silico* experiments that suggest a flexible domain structure (Anandan *et al.*, 2017 ▸). This offers some insights into how EptA might adopt conformational poses that are conducive to binding the different substrates, phosphatidyl­ethano­lamine and lipid A, and enabling the transfer chemistry to occur.

We propose these movements are an essential feature needed for the protein to perform its unique chemistry. The bridging helix acts like a hinge region in the structure that enables the protein to open up, exposing the soluble domain and the Zn^2+^ ion binding site near to the location of the PEA-bound Thr280 residue of the enzyme intermediate. This more open structure would provide an enzyme state that allows the larger sized lipid A substrate to access the PEA bound enzyme intermediate.

Thus, by using two different solution-based studies in conjunction with site-directed mutagenesis, a detailed molecular picture of EptA is emerging which helps us to better understand the enzyme chemistry and will be valuable in future drug design strategies for effective inhibitors of the enzyme as novel therapeutic agents to treat MDR bacterial infections. Additionally, the combination of SEC-SAXS and *ab initio* and rigid-body modelling methods described in this study provides a methodology-based framework for interrogating solution structures of other membrane protein systems solubilized in detergents or nanodiscs.

## Supplementary Material

Supporting figures and tables. DOI: 10.1107/S2052252521005613/jt5059sup1.pdf

## Figures and Tables

**Table 1:** Statistics for the small-angle X-ray scattering data analysis and modelling of EptA in different detergent micelles

Structure parameters	EptA in DDM micelles	EptA in DPC micelles
Gunier analysis		
*I*(0) (cm^−1^)	0.013 ± 1.9 × 10^−4^	0.0052 ± 3.3 × 10^−5^
*R*_g_ (Å)	41.96 ± 0.71	45.78 ± 0.40
*sR*_g_ limits	0.60 ± 1.30	0.40 ± 1.35
*P*(*r*) analysis		
*I*(0) (cm^−1^)	0.013 ± 0.9 × 10^−4^	0.0052 ± 2.8 × 10^−5^
*R*_g_ from *P*(*r*) (Å)	41.45 ± 0.01	48.14 ± 0.01
*D*_max_ (Å)	128.9	171.06
*s* range (Å^−1^)	0.01–0.2	0.005–0.21
		
Molecular weight estimation		
SEC-SAXS (kDa)	200.25 to 218.51	125.51 to 136.37
SEC-MALS (kDa)	160 ± 8.0	109 ± 3.4
		
Atomistic modelling and model-fitting results		
*MEMPROT* (detergent torus dimensions)		
Height (*a*) (Å)	31	24.7
Cross section (Å)		
Major axis (*b*.*e*)	34.7	32.9
Minor axis (*b*/*e*)	15.4	16.3
Ellipticity	1.5	1.42
Thickness of the hydro­philic layer (*t*) (Å)	4.8	3.7
Angle of rotation of corona around *z* axis (ω) (°)	110	0
χ^2^	1.259	6.943
		
*DADIMODO*		
No. of models generated	N/A	5
χ^2^ range for 5 models	N/A	1.05 to 1.07
*MPBuilder* (detergent corona dimensions)		
No. of detergent molecules added using *PACKMOL* routine	225	160
Bilayer height (Å)	45	44
Cross section (Å)		
Major axis	64	47
Minor axis	37.5	40
Ellipticity	1.7	1.2
		
*CORAL*		
Flexible residues	1–7, 211–230, 544–550	1–7, 211–230, 544–550
*s* range for modelling (Å^−1^)	0.01–0.32	0.006–0.288
χ^2^, *P* value	1.18, 0.00	1.84, 0.16
		
*EOM*		
Flexible residues	1–7, 211–230, 544–550	1–7, 211–230, 544–550
*s* range for modelling (Å^−1^)	0.01–0.10	0.01–0.10
χ^2^, *P* value	0.26, 0.20	0.99, 0.85

**Table 2:** Summary of the Stern–Volmer constants (*K*_SV_) obtained from the intrinsic tryptophan fluorescence quenching of EptA and mutants with iodide

		*K*_SV_† (M^−1^)	
Detergent		DDM	DPC
EptA_WT_		0.47 ± 0.02	0.80 ± 0.11‡
EptA_Trp126/148Phe_		0.30 ± 0.04	1.23 ± 0.21‡
EptA_Trp126/148/207Phe_		0.22 ± 0.01	0.57 ± 0.14‡

† The mean *K*_SV_ value is shown with a 95% confidence interval.

‡ Where Stern–Volmer plots exhibited downwards curvature, *K*_SV_ values were calculated from the initial linear slope.
