# Supplementary material for: Conformational flexibility of EptA driven by an interdomain helix provides insights for enzyme–substrate recognition
Source: IUCrJ. 2021 Jul 15;8(Pt 5):732–46. doi: 10.1107/S2052252521005613 (PMC8420757; doi:10.1107/S2052252521005613)
Supplement: Supplementary file 1 [file m-08-00732-sup1.pdf]

IUCrJ

Volume 8 (2021)

Supporting information for article:

Conformational flexibility of EptA driven by an interdomain helix provides insights for enzyme–substrate recognition

Anandhi Anandan, Nicholas W. Dunstan, Timothy M. Ryan, Haydyn D. T. Mertens, Katherine Y. L. Lim, Genevieve L. Evans, Charlene M. Kahler and Alice Vrielink

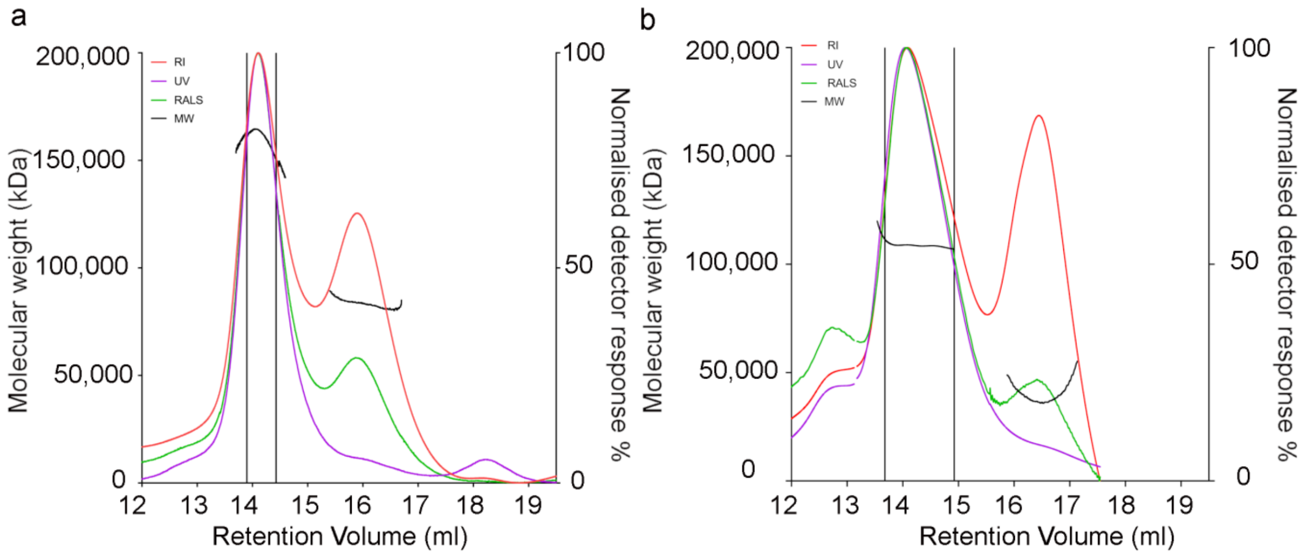

Figure S1. SEC-MALS analysis for EptA in different detergent micelles. SEC MALS profile for EptA (a) in DDM micelles and (b) in DPC micelles. The UV absorption signal is shown in purple, the refractive index signal is shown in red and the right-angle scattering signal is shown in green. The black vertical bars indicate the peak limits used for Malvern co-polymer analysis.

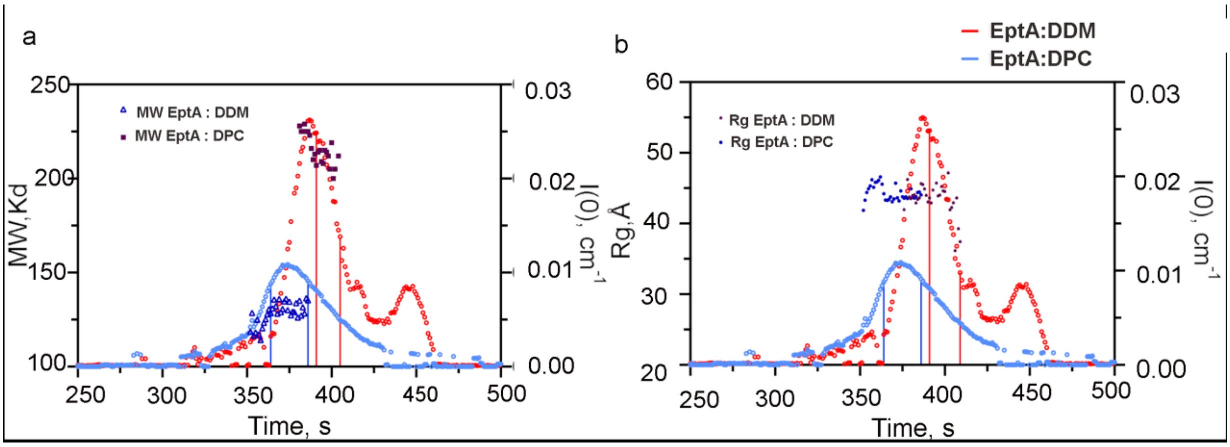

Figure S2. Molecular weight analyses of EptA in different detergent micelles from the SAXS scattering curves. (a) Plot showing I(0) (red curve for the EptA:DDM complex and blue curve for the EptA:DPC complex) and molecular weight (maroon squares for the EptA:DDM complex and blue triangles for the EptA:DPC complex) as a function of time for each SEC-SAXS run. (b) Plot showing I(0) (red dots for the EptA:DDM complex and blue dots for the EptA:DPC complex) and R_g (maroon squares for the EptA:DDM complex and blue triangles for the EptA:DPC complex) as a function of time for each SEC-SAXS run. The bars (red for the EptA:DDM complex and blue for the EptA:DPC complex) indicate the peak limits used for further SAX analyses.

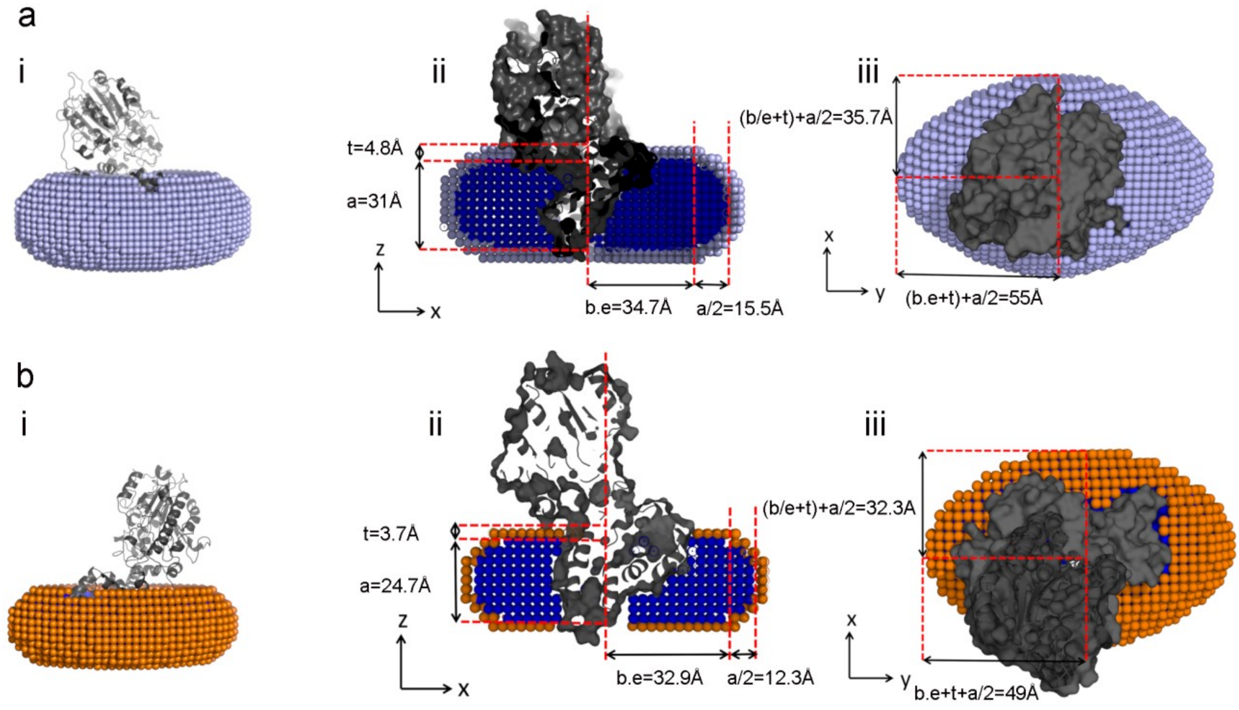

Figure S3. Models for the PDC complex based on MEMPROT analysis. Torus dimensions of MEMPROT generated models for (a) the DDM corona for the EptA:DDM complex and (b) the DPC corona for the EptA:DPC complex using the PDB file 5FGN as the protein model. Representative MEMPROT generated model for (a)(i) the EptA:DDM complex and (b)(i) the EptA:DPC complex. The atomic structure of EptA is represented in cartoon mode and colored grey. The DDM corona around EptA is represented as pale blue spheres and the DPC corona around EptA is represented as orange spheres. Cross sectional view of (a)(ii) the DDM corona and (b)(ii) the DPC corona with the height of the hydrophobic core (‘*a’*) modeled as dark blue spheres for both the DDM corona and the DPC corona. The hydrophilic shell of thickness (‘*t’*) is represented as pale blue spheres for the DDM corona and orange spheres for the DPC corona. Top view of the modeled elliptical torus depicting the minor (‘2×(*b/e+t)+a/2’*) and a major (‘2×(*b.e+t)+a/2’*) axis for (a)(iii) the DDM corona and (b)(iii) the DPC corona.

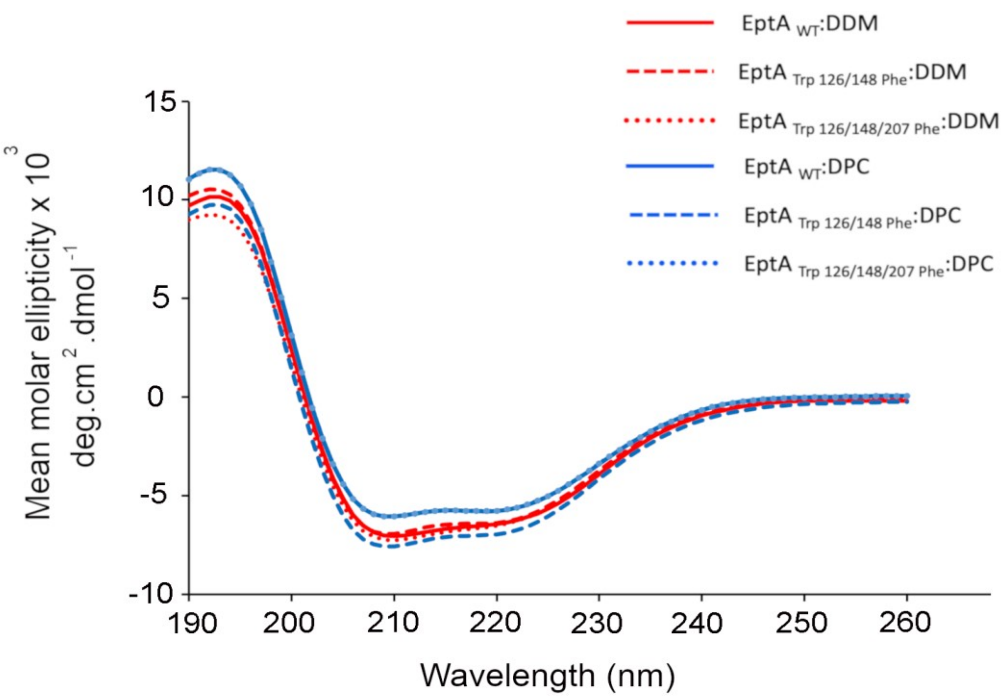

Figure S4. CD spectra of EptA in DDM micelles and DPC micelles. The red colored curves represent the enzymes solubilized and purified in DDM micelles and the blue colored curves represent the enzymes solubilized and purified in DPC micelles.

Table S1. Primers used for construction of the *eptA*:His6 tryptophan mutants, with codons used to introduce tryptophan to phenylalanine mutations shown in bold and underlined.

Primer	Sequence	Purpose
KAP674	5'-TAGAGGATCGAGATCTCGATCCCG-3'	Forward primer annealing to pET28a (+), used in Round 1 of SOE PCR to amplify fragments of *eptA*:His6
KAP675	5'-GAGGTGCCGTAAAGCACTAAATCG-3'	Reverse primer annealing to pET28a (+), used in Round 1 of SOE PCR to amplify fragments of *eptA*:His6
KAP725	5'-CCCCGGGATCCGATATCTTTTGTTTAACTTTAAGAAGG-3'	Forward primer annealing to pET28a (+), used in Round 2 of SOE PCR to 'sew' mutated fragments together
KAP726	5'-CCCGGTCGACGATATCTTCCTTTCGGGCTTTGTTAGC-3'	Reverse primer annealing to pET28a (+), used in Round 2 of SOE PCR to 'sew' mutated fragments together
KAP757	5'-GCTATGTGCTG**<u>TTC</u>**ATTGTATGTTTGG-3'	Forward primer used in Round 1 of SOE PCR to introduce the W126F mutation into *eptA*:His6
KAP758	5'-CCAAACATACAAT**<u>GAA</u>**CAGCACATAGC-3'	Reverse primer used in Round 1 of SOE PCR to introduce the W126F mutation into *eptA*:His6
KAP759	5'-GGTTAAATACCGCGTT**<u>TTC</u>**TATAAGG-3'	Forward primer used in Round 1 of SOE PCR to introduce the W148F mutation into *eptA*:His6
KAP760	5'-CCTTATA**<u>GAA</u>**AACGCGGTATTTAACC-3'	Reverse primer used in Round 1 of SOE PCR to introduce the W148F mutation into *eptA*:His6
KAP761	5'-CGAAATACAAAGAT**<u>TTC</u>**AAGCGTTCC-3'	Forward primer used in Round 1 of SOE PCR to introduce the W207F mutation into *eptA*:His6
KAP762	5'-GGAACGCTT**<u>GAA</u>**ATCTTTGTATTTCG-3'	Reverse primer used in Round 1 of SOE PCR to introduce the W207F mutation into *eptA*:His6

Table S2. SEC-MALS and small angle X-ray scattering data collection and data reduction statistics

	**EptA in DDM micelles**	**EptA in DPC micelles**
**Sample details**		
Organism	*Neisseria meningitidis*	
Source	Recombinant protein from *E. coli* (BL21 (DE3) pLysS)	
**SEC-MALS**		
SEC-MALS column	S200 10/300 increase column (GE Healthcare)	
Loading concentration (mg ml^{-1})	1.0	1.0
Injection volume (µl)	100	100
Flow rate (ml min^{-1})	0.3	0.3
Buffer	50mM Hepes, pH 7.0, 100mM NaCl and 3XCMC DDM	50mM Hepes, pH 7.0, 100mM NaCl and 3XCMC DPC
**SAXS**		
SEC- SAX column	S200 10/300 increase column (GE Healthcare)	
Loading concentration (mg ml^{-1})	7.0	7.0
Injection volume (µl)	50	50
Flow rate (ml min^{-1})	0.2	0.4
Buffer	50mM Hepes, pH 7.0, 100mM NaCl, 0.1% sodium azide and 4XCMC DDM	50mM Hepes, pH 7.0, 100mM NaCl, 0.1% sodium azide and 3XCMC DPC
**Data collection**		
Beamline	SAXS/WAXS beamline, Australian Synchrotron	
Detector	Dectris-PILATUS3 1M	Dectris-PILATUS2 1M
Beam geometry (µm)	230 x 400	
Wavelength (Å)	1.0332	
q range (Å)	0.01 – 3.0	
Camera length (m)	1.5	2.683
Exposure time (s)	5.05	1.05
Sample configuration	SEC-SAXS with sheath flow cell	
Sample temperature (ºC)	10	
**Software employed for SAX data reduction, analysis and interpretation**		
Sax data reduction	I(q) versus q using ScatterBrain 2.82 (https://asuserwiki.atlassian.net/wiki/spaces/UO/pages/358875147/scatterBrain) solvent subtraction using Chromixs (*ATSAS 3.0*, (Franke et al., 2017))	
Extinction coefficient estimate	*ProtParam* (Elisabeth Gasteiger et al., 2005)	
Basic analysis: Guinier	*PRIMUS* from *ATSAS 3.0* (Petoukhov et al., 2012)	
Structure modelling	Memprot (Perez & Koutsioubas, 2015)	Memprot
		DADIMODO (Evrard et al., 2011)
	MPbuilder (Molodenskiy et al., 2021)	MPbuilder
	CORAL	CORAL
**Input parameters for model building**		
Crystal structure	PDB entry: 5FGN	
Residues assigned as flexible region	175 to 230	
**Detergent properties**		
Mol Wt (KDa)	511	351
CMC (mM)	0.17	1.5
Aggregation number	78 - 149	50 - 60

Table S3. Minimum inhibitory concentrations (MIC) for the polymyxin B phenotypic assay on $EptA_{Trp126/148Phe}$, $EptA_{Trp126/148/207Phe}$ and WT EptA

**Strain and genotype**	**MIC (µg/ml)**
NMB	384
NMBΔ*eptA*	0.19
NMBΔ*eptA* expressing WT EptA::His6 from the shuttle vector	384
NMBΔ*eptA* expressing the shuttle vector only	0.19
NMBΔ*eptA* expressing $EptA_{Trp126/148Phe}$::His6 from the shuttle vector	384
NMBΔ*eptA* expressing $EptA_{Trp126/148/207Phe}$::His6 from the shuttle vector	384
